# RUNX1/CD44 axis regulates the proliferation, migration, and immunotherapy of gliomas: A single-cell sequencing analysis

**DOI:** 10.3389/fimmu.2023.1086280

**Published:** 2023-01-26

**Authors:** Hao Zhang, Hui Cao, Hong Luo, Nan Zhang, Zeyu Wang, Ziyu Dai, Wantao Wu, Guodong Liu, Zongyi Xie, Quan Cheng, Yuan Cheng

**Affiliations:** ^1^ Department of Neurosurgery, The Second Affiliated Hospital, Chongqing Medical University, Chongqing, China; ^2^ Department of Psychiatry, Brain Hospital of Hunan Province, The Second People’s Hospital of Hunan Province, Changsha, China; ^3^ The School of Clinical Medicine, Hunan University of Chinese Medicine, Changsha, China; ^4^ College of Life Science and Technology, Huazhong University of Science and Technology, Wuhan, China; ^5^ Department of Neurosurgery, Xiangya Hospital, Central South University, Changsha, China; ^6^ National Clinical Research Center for Geriatric Disorders, Xiangya Hospital, Central South University, Changsha, China; ^7^ Department of Oncology, Xiangya Hospital, Central South University, Changsha, China

**Keywords:** CD44, RUNX1, PD-L1, immunotherapy, glioma

## Abstract

**Background:**

Glioma is one of the most common, primary, and lethal adult brain tumors because of its extreme aggressiveness and poor prognosis. Several recent studies relevant to the immune function of CD44, a transmembrane glycoprotein as a significant hyaluronic acid receptor, have achieved great success, revealing the critical role of CD44 in immune infiltration in gliomas. The overexpression of CD44 has been verified to correlate with cancer aggressiveness and migration, while the clinical and immune features of CD44 expression have not yet been thoroughly characterized in gliomas.

**Methods:**

Molecular and clinical data of glioma collected from publicly available genomic databases were analyzed.

**Results:**

CD44 was up-expressed in malignant gliomas, notably in the 1p/19q non-codeletion cases, isocitrate dehydrogenase (IDH) wild-type, and mesenchymal subtypes in GBM samples. CD44 expression level strongly correlates with stromal and immune cells, mainly infiltrating the glioma microenvironment by single-cell sequencing analysis. Meanwhile, CD44 can be a promising biomarker in predicting immunotherapy responses and mediating the expression of PD-L1. Finally, RUNX1/CD44 axis could promote the proliferation and migration of gliomas.

**Conclusions:**

Therefore, CD44 was responsible for glioma growth and progression. It could potentially lead to a novel target for glioma immunotherapy or a prognostic biomarker.

## Introduction

Gliomas are the most common primary, lethal brain tumors in adults (1). The World Health Organization’s classification of gliomas classified central nervous system tumors into four different grades, and the grade IV glioblastoma multiforme (GBM) is the most devastating, malignant, and incurable astrocytic glioma (1, 2). The molecular diagnosis and classification of gliomas emphasized that the mutational status of isocitrate dehydrogenase (IDH) should be considered as the critical biomarker of malignancy. Except for IDH status, O-6-methylguanine DNA methyltransferase (MGMT) methylation is hitherto regarded as another significantly prognostic biomarker. Other markers are merely related to grade and further estimate prognosis, such as CDKN2A/B homozygous deletion in IDH-mutant astrocytoma, as well as 1p/19q co-deleted, TERT promoter mutation, EGFR amplification or mutation, and +7/−10 copy number changes in IDH-wildtype diffuse astrocytoma (3).

Multiple approaches and therapies have been explored to increase the efficiency of care and try to prolong survival in glioma patients over the recent several years, such as traditional aggressive surgery, radiation therapies, and chemotherapies, plus available new cocktails of drugs (4, 5). At the same time, the life expectancy of patients with GBM is fewer than 15 months after diagnosis with the standard of care (1, 6). To improve this situation, new therapeutic strategies are desperately needed to target the critical signaling pathways involved in gliomas and reduce their mortality and morbidity (7, 8).

Immunotherapy is extremely attractive for further exploration as a new therapeutic approach due to the emerging evidence from recent discoveries and studies demonstrating long-lasting tumor remission with fewer side effects. For example, PD-1, the typical immune checkpoint and the most dominant target molecule in cancer immunotherapy, has now aroused the majority of attention. Yet, despite limited achievements in treating several solid tumors with PD-1 antibody inhibition, such as the esophagus or non-small cell lung cancer, few advances have been found in glioblastoma (9, 10). Another inhibitory immune pathway is cytotoxic T lymphocyte-associated antigen 4 (CTLA-4), which is also viewed as a critical role in the aggressiveness of gliomas. The anti-therapy aimed at CTLA-4 seems less successful in gliomas because of complicated reasons, including the blood-brain barrier and a lack of guiding biomarkers of the blockade (11). Although many scientists have been trying different strategies, such as peptides and dendritic cell (DC) vaccines, there was not much success in clinical trials, especially the increased overall survival (OS) with good life quality. More efforts still are needed in this area (12).

CD44, also known as cluster determinant 44, is a single-pass transmembrane glycoprotein comprised of four domains: cytoplasmic, transmembrane, stem structure, and amino-terminal. And its amino-terminal domain can be the docking site for the extracellular matrix (ECM) components like hyaluronic acid, which is the main glycosaminoglycan in the brain’s ECM and play a critical factor in glioma invasion. The stem structure works as a linking part between the transmembrane and amino-terminal domains, and its function has remained unknown. The function of the transmembrane region of CD44 was expected to be associated with lipid rafts. Significantly, the cytoplasmic domain can interact with proteins involved in motility and cell adhesion, including ankyrin and ERM proteins. In addition, this region also can interact with various regulatory and adaptor molecules implicated in cell signaling, which is the basis of CD44 as a pivotal role in MMP-mediated matrix degradation, tumor development, migration, and invasion (13).

Previous research has provided evidence of high CD44 expression in different tumors, such as head and neck cancer, lung cancer, breast cancer, etc. (14–16). However, the mechanism of CD44’s involvement in cancer metastasis remains unknown. A finding based on bone metastatic cancer stem cells has revealed that the interaction with HA may be the possible reason, making it a potential target for drug intervention (17). Some studies also illustrated that several variant isoforms of CD44 are directly linked with the signal pathway of cancer cell migration and invasion (18, 19). A few studies have focused on the molecular and clinical pattern of CD44 expression in gliomas. CD44 serves as a hazardous marker in grade II/III gliomas (20). CD44 was reported to be associated with glial dynamics in the tumor microenvironment (21). MiR-373/miR-520s-CD44 axis could significantly inhibit the growth and invasion of GBM (22). CD44 expressed by myeloid cells was found to promote the invasion of glioma (23). EHD1 was revealed to promote the cancer stem cell (CSC)-like traits of glioma *via* interacting with CD44 and suppressing CD44 degradation (24). Besides, CD44 was recently demonstrated to be associated with the M2-polarization of tumor-associated macrophages and immunosuppression of glioma (25). Notably, spatially resolved proteomic profiling recently identified CD44 as a biomarker associated with anti-PD-1 sensitivity in advanced non-small-cell lung cancer (26). Osteopontin/CD44 could control CD8+ T cell activation and tumor immune evasion (27). Therefore, CD44 was proposed as a potential regulator of macrophage and immunotherapy in gliomas.

In this study, we tried to figure out the role of CD44 expression and thoroughly investigated the molecular pattern of CD44 as well as the clinical association with LGG and GBM. By researching the whole database, our study is the first complete one that characterizes and delineates the expression of CD44 in LGG and GBM from molecular and clinical views. CD44 was found as an important regulator of macrophage and immunotherapy in gliomas. Besides, RUNX1/CD44 axis critically mediated the proliferation and migration of gliomas. Understanding the CD44 expression pattern and features in glioma better will assist in different optimal strategies for glioma therapies.

## Materials and methods

### Data collection

The Cancer Genome Atlas (TCGA), Chinese Glioma Genome Atlas (CGGA), and Gene Expression Omnibus (GEO) datasets were mainly used for the follow-up study. Ivy Glioblastoma Atlas Project (GAP) dataset (28) and Gill dataset (29) were included.

### Mutation analysis

Copy number alternations (CNAs) associated with CD44 expression were analyzed using GISTIC 2.0.

### Immune analysis

The immune infiltrating cells were quantified by MCPcounter algorithm (30), ssGSEA algorithm (31), and TIMER algorithm (32). ESTIMATE algorithm (33) was used for calculating the microenvironment scores. The cancer immunity cycle was conducted using get set variation analysis (GSVA) (34, 35). 114 metabolic pathways from the previous literature were quantified using GSVA (36).

### Single-cell RNA sequencing analysis

The scRNA-seq matrix of GSE138794 (37) was conducted using the R package Seurat. The detailed procedure was reported in our previous study (38). “FeaturePlot”, “Dimplot”, and “VlnPlot” were applied to visualize the expression of CD44. Single-cell pseudotime trajectories analysis was processed using the R package Monocle (39). Gene ontology (GO) and Kyoto Encyclopedia of Genes and Genomes (KEGG) enrichment analyses were performed.

### Western blotting assay

The western blotting assay evaluated the expression level of RUNX1, CD44, PD-L1, and β-actin. Anti-RUNX1 (ab240639, Rabbit, 1:1000, Abcam, UK), anti-CD44 (15675-1-AP, Rabbit, 1:2000, Proteintech, China), anti-PD-L1 (66248-1-Ig, Mouse, 1:2000, Proteintech, China), and anti-β-actin (66009-1-Ig, Mouse, 1:5000, Proteintech, China) were applied as the primary antibody. HRP goat anti-mouse IgG (SA00001-1, Mouse, 1:5000, Proteintech, China) and HRP goat anti-rabbit IgG (SA00001-2, Rabbit, 1:6000, Proteintech, China) were applied as the secondary antibody.

### RT-qPCR assay

The primers of GADPH (F ACAGCCTCAAGATCATCAGC; R GGTCATGAGTCCTTCCACGAT) and CD44 (F CAGCTCATACCAGCCATCCA; R TGGGGTGTGAGATTGGGTTG) were designed by the primer 5.0. Total RNAs were extracted and reversely transcribed into cDNA by HiScript Q RT SuperMix for RT-qPCR. GADPH and CD44 expression levels were conducted using 2-ΔΔCT.

### Cell culture and transfection

U251 cells were cultured in Dulbecco’s modified eagle medium (DMEM), with 10% fetal bovine serum (FBS) in the saturated humidity incubator (37°C, 5% CO2). The RUNX1 specific primers for overexpression (OV) plasmid were as follows: F CACACTGGACTAGTGGATCCCGCCACCATGGCTTCAGACAGCATATTTG; R AGTCACTTAAGCTTGGTACGTAGGGCCTCCACACGGCCTCCTC. The RUNX1-OV plasmid was transfected into U251 cells *via* Lipofectamine 2000. The short hairpin (sh)-RNA target sequences of CD44 (93684-1, GACCTCTGCAAGGCTTTCAAT; 93685-1, CTGCCGCTTTGCAGGTGTATT; 93686-11, GAGCATCGGATTTGAGACCTG) were used for constructing the lentivirus. RUNX1-OV and RUNX1-NC groups of U251 cells were transfected with a lentivirus vector and cultured in DMEM with puromycin for stable CD44-knockdown U251 cells.

### Cell counting Kit-8 assay

The RUNX1-NC, RUNX1-OV, RUNX1-OV+CD44-shRNA, and RUNX1-NC+CD44-shRNA groups of U251 cells were seeded in 24-well plates, with a density of 1×10^4^ cells per well. 30 ul CCK8 reagent was diluted in 270 ul DMEM and subsequently added to each well. Later, U251 cells were cultured for 0h, 24h, 48h, and 72h. The absorbance was measured at 450 nm.

### Clone formation assay

The RUNX1-NC, RUNX1-OV, RUNX1-OV+CD44-shRNA, and RUNX1-NC+CD44-shRNA groups of U251 cells were plated in 6-well plates for 2 weeks, with a density of 200 cells per well. The colonies were fixed with 4% methanol (1 ml per well) for 15 min and stained with 0.5% crystal violet. Representative images of each well were collected.

### Transwell assay

500 ul DMEM with 10% FBS was added to the lower chamber of a 6-well plate. The RUNX1-NC, RUNX1-OV, RUNX1-OV+CD44-shRNA, and RUNX1-NC+CD44-shRNA groups of U251 cells were digested and resuspended with a density of 2x10^6^ cells/ml using DMEM. 100 ul U251 cells were added to the upper chamber and cultured for 48h. The upper chamber was stained with 0.5% crystal violet for 5 min. Representative images of each well were collected.

### IHC staining

The sections of different grades of gliomas were collected. Anti-CD44 (15675-1-AP, Rabbit, 1:500, Proteintech, China) was used as the primary antibody. The horseradish peroxidase-conjugated antibody (ZSGB-BIO, PV-9000, China) was used as the secondary antibody. The sections were stained with 3, 30-diaminobenzidine tetrahydrochloride (DAB) and counterstained with hematoxylin.

### Statistical analysis

Spearman or Pearson correlation analysis was used to evaluate the correlations between continuous variables. For normally distributed variables, significant quantitative differences between and among groups were determined by a two‐tailed t-test or one‐way ANOVA, respectively. For nonnormally distributed variables, significant quantitative differences between and among groups were determined by a Wilcoxon test or a Kruskal–Wallis test, respectively. All statistical analyses were conducted by the R project (https://www.r-project.org/). It is considered to be statistically significant when P-values < 0.05. All tests were two-sided.

## Result

### Up-regulated CD44 expression in gliomas

We delineated the pattern of CD44 mRNA expression levels regarding disparate WHO-grade gliomas by evaluating data from publicly available databases. Firstly, we checked the expression pattern of CD44 in WHO grades, and then subtypes were divided into LGG and GBM. Not surprisingly, the CD44 expression was observed to be significantly increased in higher WHO grades according to datasets from the CGGA and TCGA (**Figure S1A**). Also, compared to LGG patients, GBM groups showed higher levels of CD44 expression, which demonstrated an adverse role of CD44 in the proliferation and aggressive progress of gliomas. (**Figure S1B**). The representative images of CD44 in immunohistochemistry staining of different grades of glioma are shown in **Figure S1C**.

Moreover, we tested CD44 in two important gene mutation cohorts extensively used as clinical outcome prediction tools. Interestingly, the IDH-mutated gliomas, linked with better clinical outcomes (40), were associated with a lower CD44 level in pan-gliomas and GBM cases (**Figures S1D, E**). Furthermore, the ROC curve demonstrated that the value of CD44 is an effective predictor of IDH mutation in GBM and pan-gliomas cases (AUC value = 0.789; AUC value = 0.773, respectively **Figure S1F**). Similarly, analysis in 1p/19q non-codeletion pan-glioma showed up-expressed levels of CD44, which can predict the poor survival of glioma. Still, analysis in 1p/19q codeletion pan-glioma showed down-expressed levels of CD44 (**Figure S1G**). Importantly, in LGG cases, 1p/19q codeletion and IDH mutation are correlated with lower expression levels of CD44 (**Figure S1H**). At the same time, we tested the sensitivity of CD44 as a prognostic biomarker in predicting the survival of 1 year, 3 years, and 5 years, respectively. (**Figure S1I**). We found the AUC of 1 year is 0.796, the AUC of 3 years is 0.767, and the AUC of 5 years is 0.766 according to the TCGA dataset, while the AUC of 1 year is 0.633, the AUC of 3 years is 0.687, and the AUC of 5 years is 0.688 according to the CGGA dataset. In addition, CD44 was found to be lower in expression levels in methylated glioma in the TCGA dataset (**Figure S2A**). The specific methylation probe significantly associated with gliomas regarding CD44 expression is displayed in **Figure S3**. The levels of CD44 expression in the histology of glioma tissues and various cell lines are shown in **Figure S2B**.

### Characteristics, expression pattern, and distribution of CD44 in gliomas

There are three molecular categories with distinct sub-classes in human gliomas: classical (CL), proneural (PN), and mesenchymal (MS). Among these three sub-classes, subtypes of CL and MS show more aggressiveness in behavior (10, 41). To delineate the inter-tumor heterogeneity of CD44, we studied it among four molecular sub-classes according to the VERHAAK_2017 classification scheme (42). Based on the TCGA dataset, GBM and pan-gliomas of CL and MS subtypes had significantly higher expression levels of CD44 compared to subtypes of PN (**Figures S2C, E**). Furthermore, the ROC curve demonstrated that CD44 might play a role as a predictor of subtypes of CL and MS in GBM and pan-gliomas cases (AUC value = 0.833; AUC value = 0.782, **Figures S2D, F**).

To detect the intra-tumor distribution of CD44 expression in glioma tissues, we examined CD44 expression levels in disparate structures. According to the Ivy Glioblastoma Atlas Project data, in peri-necrotic zones, pseudo palisading cells around necrosis, cellular tumors, and hyperplastic blood vessels, CD44 was highly expressed (**Figure S2G**). Interestingly, in radio graphical regions, different compositions were discovered in the T1 contrast-enhancing (CE) regions, compared with the non-contrast-enhancing (NCE) margins. The results of RNA sequencing analysis illustrated that CE regions have higher CD44 expression compared to NCE or normal tissue (NT) areas (**Figure S2H**).

### Higher CD44 expression is found in glioma patients with poor survival

**Figure 1:**
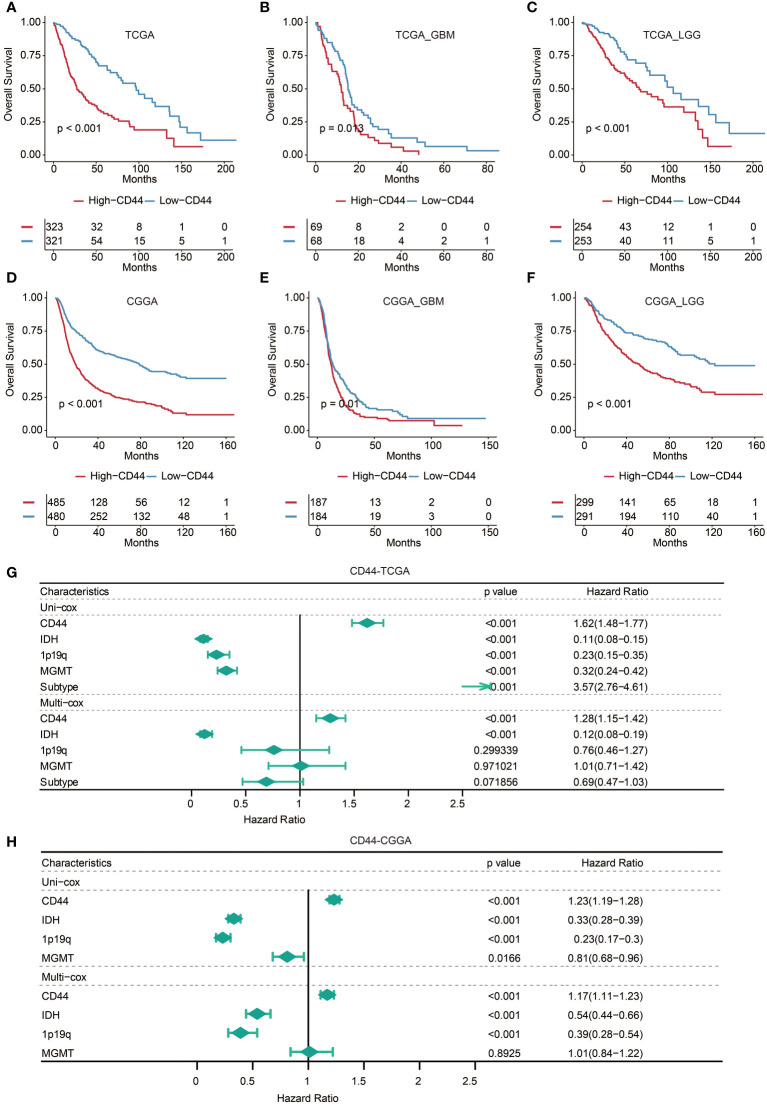
Higher CD44 expression is found in glioma patients with poor survival. Kaplan-Meier analysis of OS based on high vs. low expression of CD44 in the TCGA **(A-C)** and CGGA **(D-F)** datasets. The median value of CD44 was used as the cut-off value. P-values were obtained from the log-rank test. **(G)** Univariate and multivariate Cox regression analysis on clinical factors in the TCGA dataset. **(H)** Univariate and multivariate Cox regression analysis on clinical factors in the CGGA dataset.

Consequently, we evaluated prognostic value concerning the different CD44 levels in gliomas through Kaplan-Meier analysis. Compared to CD44^low^ patients, CD44^high^ patients showed significantly shorter OS in GBM, pan-gliomas, and LGG samples from TCGA and CGGA datasets (**Figures 1A–F**). Further, based on the univariate and multivariate Cox regression analysis, CD44 showed the potential as an independent prognostic factor with IDH, 1p19q, MGMT, and Subtype (**Figures 1G, H**).

After all our work, the prediction value of CD44 as a biomarker in clinical use still needs to be examined. We implied a prognostic nomogram model developed by CD44 expression and clinicopathological risk factors to test its prediction in the clinical prognosis of gliomas (**Figure S4A**). Nomograms, a widely used graphical statistical model in cancer, are formed by a statistical algorithm using a combination of all predictive factors that have been admitted to affect the probability of a clinical event. Calibration plots revealed nomograms’ potential to predict patients’ survival based on an ideal model (**Figure S4B**). Kaplan-Meier survival curve demonstrated the statistical significance of the survival difference between high and low-risk patients (**Figure S4C**). The AUC of 4 years is 0.865 in the TCGA dataset (**Figure S4D**).

### CD44 predicts the drug response in gliomas

PRISM and GDSC databases were used for drug prediction based on CD44 expression. The top correlated drugs in GDSC1 are shown in **Figure S5A**. The top correlated drugs in GDSC2 are demonstrated in **Figure S5B**. The top correlated drugs in PRISM were shown in **Figure S5C**. Notably, AZD6482, Bicalutamide, Bortezomib, Cyclopamine, Dasatinib, Lapatinib, Paclitaxel, Parthenolide, Pazopanib, Rapamycin, and Sorafenib were found with lower estimated IC50 in CD44^high^ glioma patients (**Figure S5D**).

### CD44 affects stromal and immune cell infiltration in gliomas

**Figure 2:**
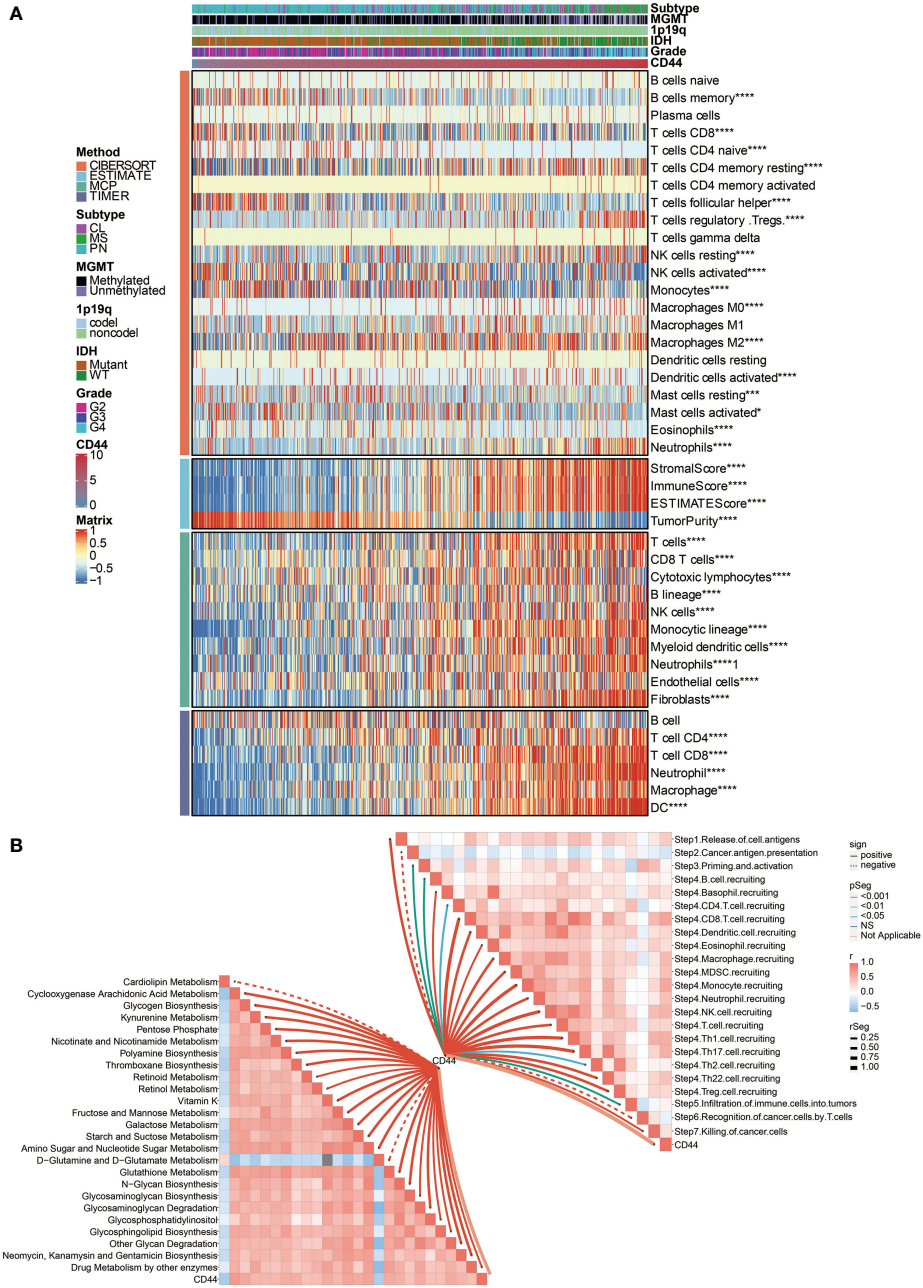
Immune processes and metabolism related to CD44 expression. **(A)** The association between CD44 and immune infiltrating cells was estimated by CIBERSORT, ESTIMATE, MCPcounter, and TIMER algorithms. **(B)** The correlation between CD44 and cancer immunity cycle and metabolic pathways. *, P< 0.05; ***, P<0.001; ****, P<0.0001.

Two central cells consist of solid tumors: stromal cells and cancer cells. At the same time, stromal cells and infiltrating immune cells build up most of the tumor microenvironment. First, the correlation between ESTIMATE scores and CD44 expression was explored. Data demonstrated a significantly positive association between CD44 expression level and stromal score, immune score, and ESTIMATE score according to the TCGA dataset (**Figure 2A**), indicating that changing stromal and infiltrating immune cells, CD44 can affect the glioma microenvironment. These results also inspired us to believe that there might be a specific role for CD44 in glioma development and migration. To further study the relationship between high CD44 levels in gliomas and tumor immunity function, we performed CIBERSORT, MCPcounter, and TIMER analyses to determine whether the typical immune cells responsible for the immune responses against tumors or participating in the inflammatory activity are related to high CD44 expression. We discovered the positive association between CD44 and multiple important infiltrating immune cell types like monocytes, macrophages, Myeloid-derived suppressor cells (MDSC), neutrophils, and natural killer (NK) cells in the TCGA dataset (**Figure 2A**). Altogether, our data revealed that CD44^high^ patients tended to increase immune cells infiltrating within their tumor microenvironment.

### CD44 is associated with immune processes and metabolism

Then we turned to our most exciting part and evaluated the possible immune-related functions of CD44 in glioma cell growth in the TCGA dataset. Based on the cancer immunity cycle, CD44 was positively associated with the release of cell antigens, priming and activation, recruiting of CD4 T cell, CD8 T cell, Th1 cell, Th2 cell, Th17 cell, Th22 cell, Treg cell, B cell, eosinophil, neutrophil, dendritic cell, macrophage, NK cell, and the infiltration of multiple immune cells (**Figure 2B**).

Besides, CD44 was positively associated with several metabolic pathways, including glycogen biosynthesis, galactose metabolism, glycosaminoglycan biosynthesis, N-glycan biosynthesis, and starch and suctose metabolism (**Figure 2B**), indicating a highly activated tumor microenvironment.

### Neoplastic cells and macrophages show up-regulated CD44 in scRNA-seq of gliomas

**Figure 3:**
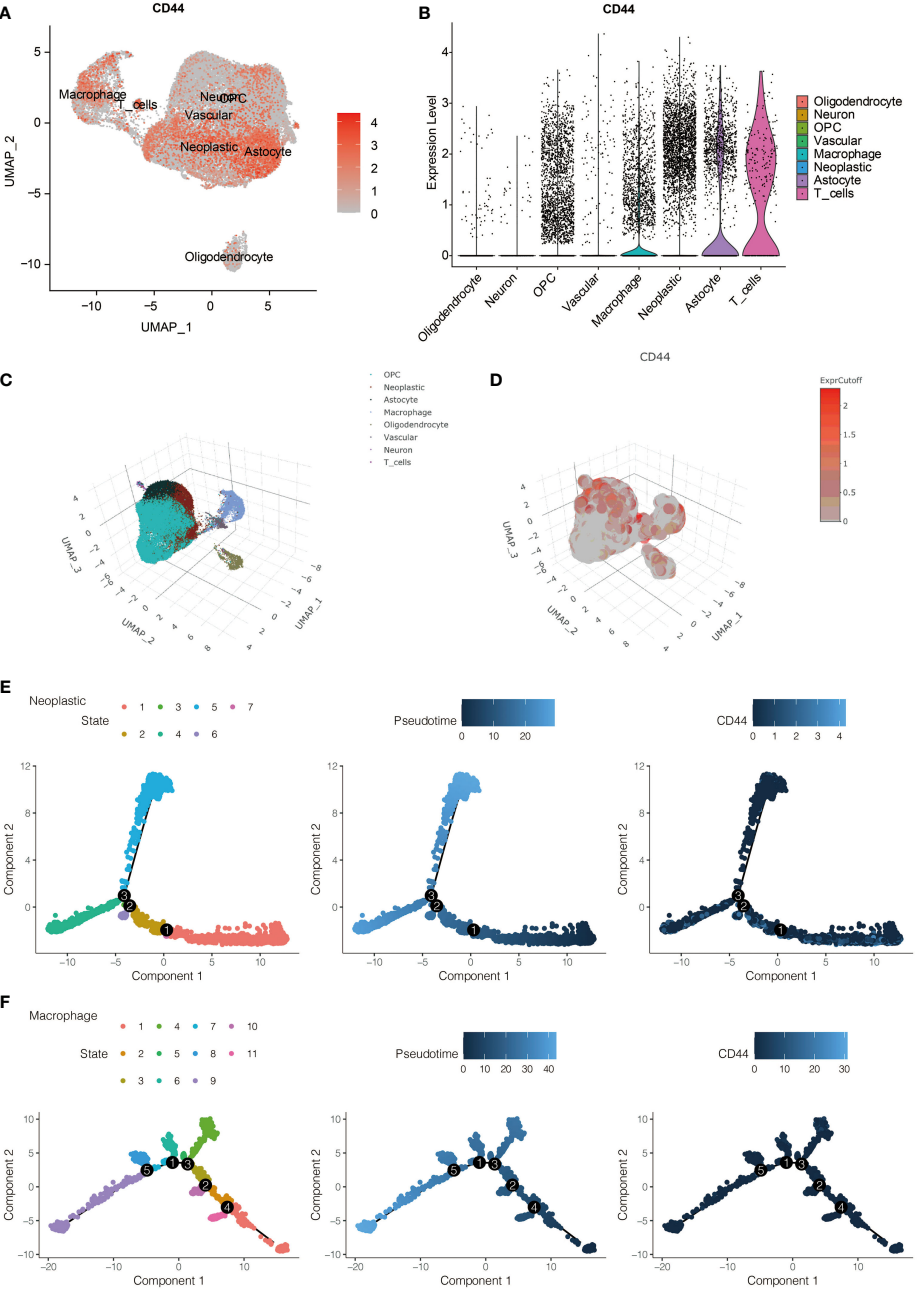
The expression pattern of CD44 at the scRNA-seq level. **(A)** The expression distribution of CD44 in eight types of identified cells. **(B)** The expression level of CD44 in eight types of identified cells. **(C)** Three-dimension plot of eight types of identified cells. **(D)** Three-dimension plot of the expression level of CD44 in eight types of identified cells. **(E)** The pseudotime trajectory analysis of glioma cells. **(F)** The pseudotime trajectory analysis of macrophages.

To further evaluate the role of CD44 in immune infiltrating, the expression of CD44 in gliomas was also analyzed by scRNA-seq. The expression of CD44 in all eight identified cell types is visualized in **Figure 3A**. CD44 was richly expressed in neoplastic cells, macrophages, astrocytes, and T cells (**Figure 3B**). Additionally, the distribution of eight cell clusters was visualized by a three-dimension plot (**Figure 3C**). The correlation of CD44 and neoplastic cells, macrophages, astrocytes, and T cells is confirmed (**Figure 3D**).

**Figure 4:**
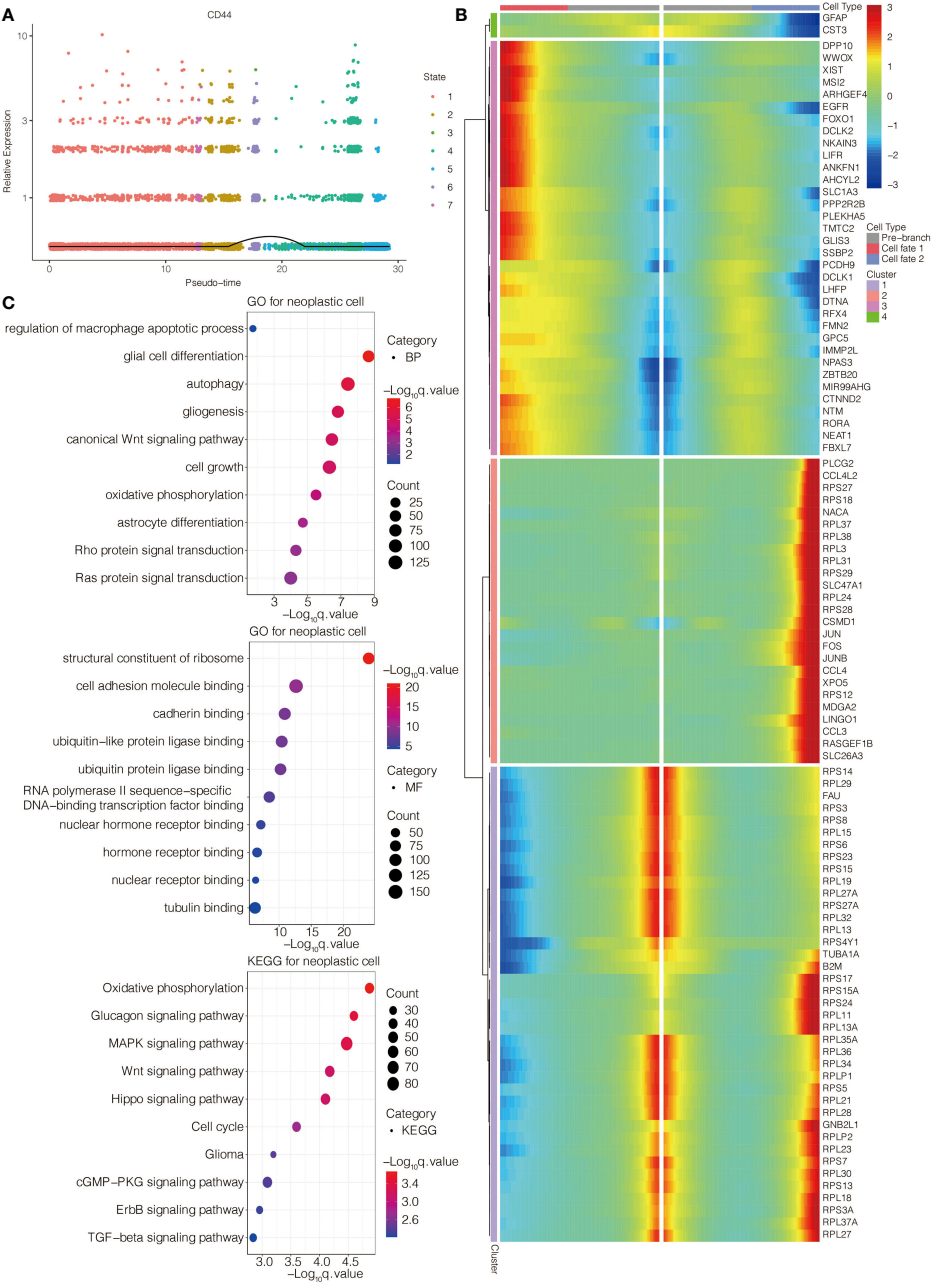
Function annotation of CD44 in glioma cells at the scRNA-seq level. **(A)** The expression level of CD44 regarding the pseudotime and cell state changes. **(B)** The gene expression pattern of 100 genes with branch-dependent expression for branch point 3. **(C)** GO and KEGG enrichment analysis.

**Figure 5:**
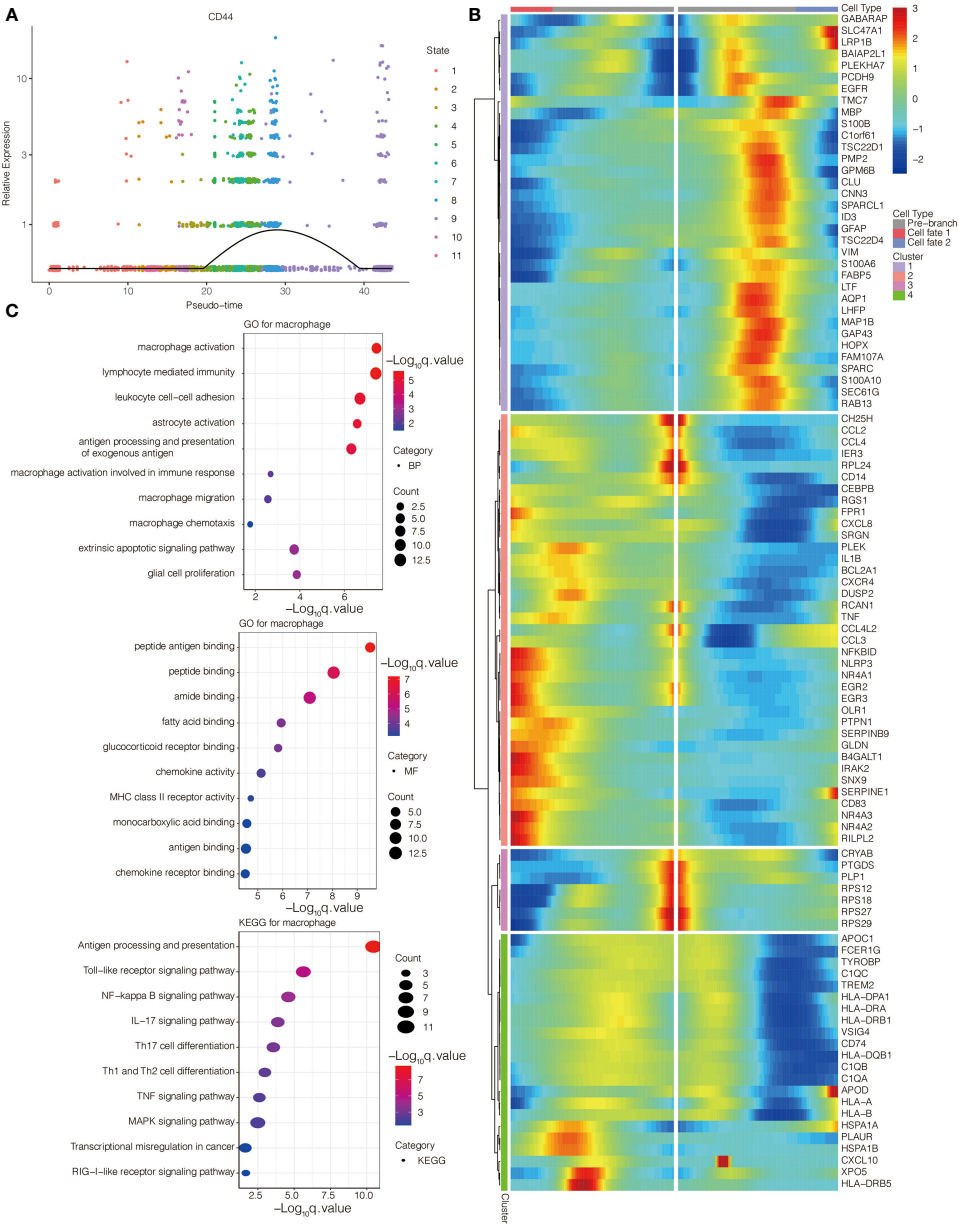
Function annotation of CD44 in macrophages at the scRNA-seq level. **(A)** The expression level of CD44 regarding the pseudotime and cell state changes. **(B)** The gene expression pattern of 100 genes with branch-dependent expression for branch point 3. **(C)** GO and KEGG enrichment analysis.

The single-cell pseudotime trajectories and functional annotations of neoplastic cells and macrophages were explored. In neoplastic cells, a trajectory was reconstructed, containing three branch points and grouped cells into seven states (**Figure 3E**). In macrophages, a trajectory was reconstructed, containing five branch points and grouped cells into eleven states (**Figure 3F**). The relative expression level of CD44 in the cell states from neoplastic cells and macrophages is presented in **Figures 4A**, **5A**, respectively. 100 genes were further identified with branch-dependent expression for branch point 3 of neoplastic cells. The expression of the different genes before and after branch point 3 and related clustering are presented in **Figure 4B**. Moreover, 100 genes that differentially expressed with branch-dependent expression for branch point 3 of macrophages were also confirmed (**Figure 5B**). Finally, in regards to CD44 in neoplastic cells and macrophages, the results of GO enrichment analysis and KEGG pathway analysis was presented in **Figures 4C**, **5C**.

### RUNX1/CD44 axis mediates the proliferation and migration of glioma

**Figure 6:**
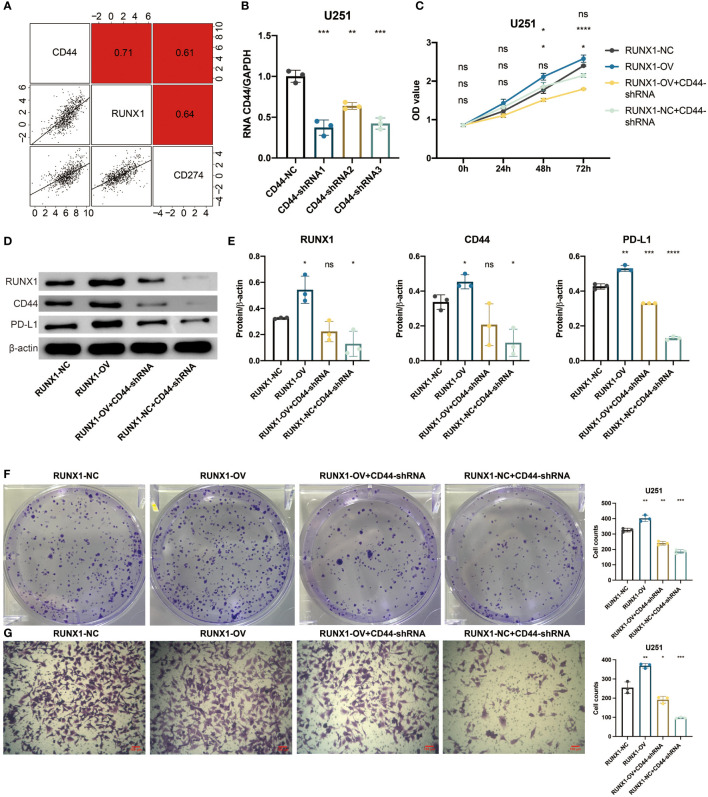
RUNX1/CD44 axis mediates proliferation and migration of glioma. **(A)** Correlation between CD44 and RUNX1, PD-L1 in the TCGA dataset. **(B)** qPCR assay for CD44 in shRNA-transfected U251 cells. **(C)** CCK8 assay for four groups of U251 cells. **(D)** Western blotting assay for RUNX1, CD44, and PD-L1 in shRNA-transfected U251 cells. **(E)** Statistical analysis of western blotting assay for RUNX1, CD44, and PD-L1. **(F)** Clone formation assay for four groups of U251 cells. **(G)** Transwell assay for four groups of U251 cells. NS, Not Statistically Significant; *, P< 0.05; **, P<0.01; ***, P<0.001; ****, P<0.0001.

To investigate the potential mechanisms of the pathogenic role of CD44 in gliomas, in vitro validation was performed. A strong positive correlation between CD44 and RUNX1 was observed in the TCGA dataset (**Figure 6A**). Lentivirus targeting CD44 was transfected into U251 cells to select the shRNA (93684-1) with the highest knockout efficiency (**Figure 6B**). The plasmid of RUNX1 was further transfected into U251 cells for the overexpression of RUNX1. Four groups, RUNX1-NC, RUNX1-OV, RUNX1-OV+CD44-shRNA, and RUNX1-NC+CD44-shRNA, were used for the follow-up experiments. The western blotting assay revealed that the overexpression of RUNX1 promoted the expression of CD44 with statistical significance (**Figures 6D, E**). Notably, the silence of CD44 suppressed the expression of RUNX1 with statistical significance. CCK8 assay revealed that CD44 inhibition could decrease the U251 cell proliferation. At the same time, the up-regulated levels of RUNX1 alleviated the reduced accumulation of U251 cells (**Figure 6C**). Clone formation assay showed that the clone formation ability of U251 cells decreased under CD44 inhibition. In contrast, the overexpression of RUNX1 alleviated the decreased clone formation capacity of U251 cells (**Figure 6F**). Transwell assay revealed that CD44 inhibition could reduce the migration of U251 cells. In contrast, the up-regulated levels of RUNX1 alleviated the reduced migration of U251 cells (**Figure 6G**). A strong positive correlation between CD44 and PD-L1was observed in the TCGA dataset (**Figure 6A**). Furthermore, western blotting assay revealed that RUNX1/CD44 could promote the expression of PD-L1 (**Figures 6D, E**). These results suggested that RUNX1/CD44 axis could mediate the proliferation, migration, and immunotherapy of glioma.

### Immunotherapy prediction of CD44

**Figure 7:**
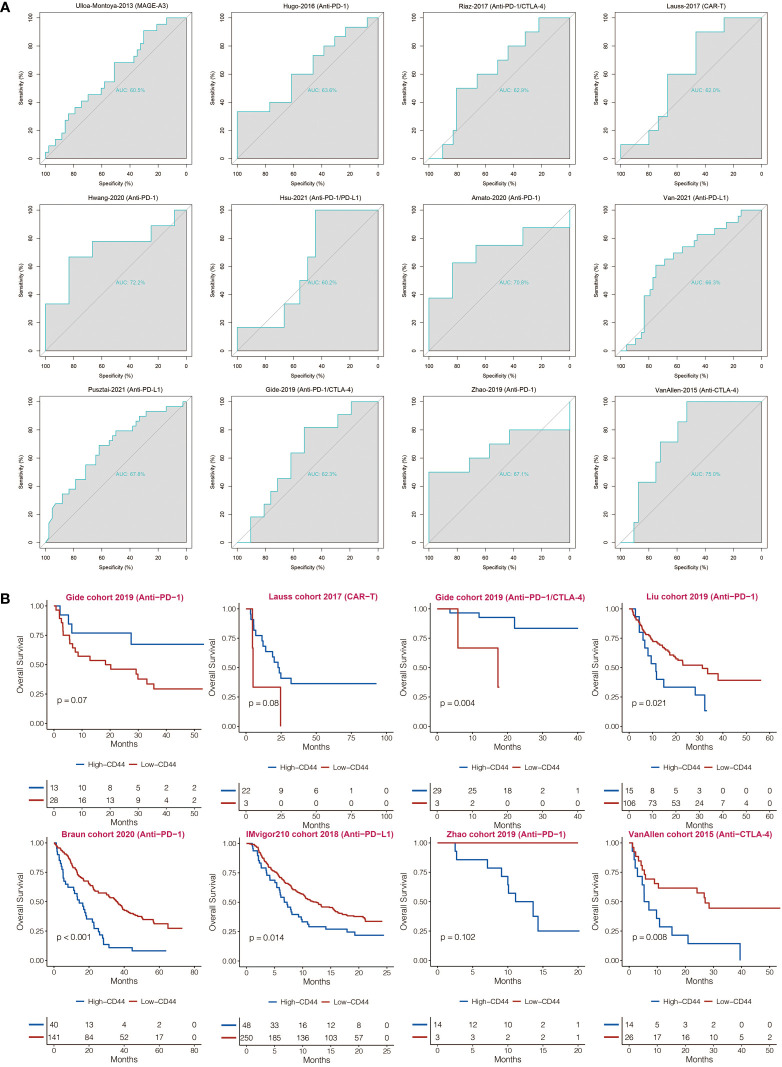
Immunotherapy prediction of CD44. **(A)** ROC curves regarding CD44 in immunotherapy cohorts. **(B)** Kaplan-Meier analysis of OS based on high vs. low expression of CD44 in immunotherapy cohorts.

CD44 was found with AUC values of larger than 0.6 in predicting the immunotherapy responses in 12 immunotherapy cohorts (**Figure 7A**). Compared to CD44^low^ patients, CD44^high^ patients showed shorter OS in the IMvigor210 cohort, Liu cohort, Braun cohort, Zhao cohort, and VanAllen cohort (**Figure 7B**). Compared to CD44^low^ patients, CD44^high^ patients showed longer OS in the Gide cohort, Lauss cohort, and Gide cohort (**Figure 7B**).

## Discussion

Many efforts have been devoted to the research of glioma curing. However, gliomas, particularly GBM, remain the most malignant brain tumor with inferior survival. Among several strategies aiming at improving treatment effects, immune therapy, eliciting an immune response, or unlocking immune checkpoints blocked by a tumor, have shown breakthroughs in many malignant tumors due to their long-lasting cancer remission with minimal damage in preclinical and clinical trials. Because of that, immune therapy has been appealing to many scientists and experts. As a principal receptor of HA, CD44 has an important influence on cell adhesion, which may affect tumor cells’ migration and aggressiveness. Therefore, a work of better understanding the CD44 feature in glioma is desperately required to develop a treatment strategy.

According to our findings by a large-scale bioinformatic analysis, we investigated the features and patterns of CD44 among gliomas. The mRNA expression levels of CD44 were significantly up-regulated in gliomas, GBM in particular. The expression levels of CD44 were higher in unmethylated glioma with IDH wildtype and glioma with 1p/19q non-codeletion. Moreover, high expression levels of CD44 were closely correlated to the CL and MS molecular subtypes, which indicated its potential to be a sensitive glioma diagnostic biomarker. CD44 was localized to peri-necrotic zones, pseudo-palisading cells around necrosis, cellular tumors, and hyperplastic blood vessels. Moreover, higher expression of CD44 has a strong relationship with poor survival. According to our exploration of the distinct genomic alternations of CD44, we found a negative association between the events of somatic mutations and CD44 expression, which indicated that CD44 expression was correlated with the aggressive glioma process. The results showed that CD44 expression was closely associated with glioma occurrence. And CD44 is vital in aiding the oncogenic process and progression of gliomas.

GBM can trigger the innate and adaptive immune systems, and various immune cell types have accumulated around the tumor. Therefore, they can come together to resist the assumptions that the human brain is immune privileged. Many types of research have proved that four kinds of immune cell types tend to help form a permissive tumor microenvironment: tumor-associated macrophagocytes, myeloid-derived suppressor cells, regulatory T cells, and cancer-associated fibroblasts, which stimulate cancer cell growth, migration, and invasion (43). Notably, few studies have focused on the immune characteristics of CD44 expression in glioma. A previous scRNA-seq analysis revealed SPP1/CD44-mediated crosstalk between macrophages and cancer cells in glioma (44). PLOD2 modulates the immune microenvironment and tumor progression of glioma, in which CD44 was the critical downstream molecule (45). Besides, CD44 was recently demonstrated to be associated with the M2-polarization of tumor-associated macrophages and immunosuppression of glioma (25). In our study, the correlation analysis between CD44 and cells in the tumor microenvironment suggested that CD44^high^ glioma cells are inclined to accumulate more inhibitive infiltrating immune cells (helper 2 T cell, regulatory T cell, and immature dendritic cell) into the tumor microenvironment. In line with our findings, GDF15 promotes the immune escape of ovarian cancer by targeting CD44 in dendritic cells (46). CD44 correlates with immune infiltrates in gastric cancer (47). Besides, IL18 increases the immune escape of gastric cancer by downregulating CD70 and maintaining CD44 (48). These results supported that CD44 had cancer-promoting activity in the immunity of gliomas through the immune microenvironment. Specifically, CD44 was found to critically mediate the activity of glioma cells and macrophages at the scRNA-seq level. The expression of CD44 could reflect the evolution direction of glioma cells towards malignancy. Besides, the expression of CD44 could reflect the evolution direction of macrophages towards immune activation.

Several immune checkpoint inhibitor treatments showed encouraging benefits in preclinical trials, such as the classical drug Ipilimumab targeting CTLA-4 and Nivolumab targeting PD-1. PD-1 is a receptor on the surface of T cells that receives suppressive signals from PD-L1 on cancer cells or APCs, which causes cytotoxic effects, cytokine production, and reduced activity of T cells (9). To make more assumptions about the clinical use of CD44 through previous effective drugs, we analyzed the relationship between CD44 and PD-L1. RUNX1/CD44 was proved to contribute to PD-L1 expression. The interaction between CD44 and PD-L1 might inspire us to combine therapy to treat gliomas, such as blocking CD44 and other immune checkpoints.

RUNX1 has been identified as a critical transcription factor regulating multiple biological processes of cancer (49). However, the potential regulatory role of RUNX1 on CD44 has not been explored. Our finding indicated that RUNX1 could potentially promote glioma proliferation and migration by up-regulating the expression of CD44. The RUNX1/CD44 axis in gliomas remained valuable for further exploration.

Taken together, our work demonstrates the significant role of CD44 in the progression and treatment of gliomas. Strikingly, CD44 probably has a more profound relationship with LGG than with GBM. Further studies are required to explore the function of CD44 and turn it into a novel site for immune-therapeutic strategy or prognostic biomarker for glioma patients.

## Data availability statement

The original contributions presented in the study are included in the article/**Supplementary Material**. Further inquiries can be directed to the corresponding authors.

## Author contributions

HZ designed and drafted the manuscript. HZ conducted the experiments. HZ, HC, NZ, ZW, ZD, WW, GL, ZX, QC, and YC revised the manuscript. All authors contributed to the article and approved the submitted version.
