# Supplementary material for: RUNX1/CD44 axis regulates the proliferation, migration, and immunotherapy of gliomas: A single-cell sequencing analysis
Source: Front Immunol. 2023 Jan 26;14:1086280. doi: 10.3389/fimmu.2023.1086280 (PMC9909339; doi:10.3389/fimmu.2023.1086280)
Supplement: Supplementary file 1 [file DataSheet_1.docx]

**Figure Legends**


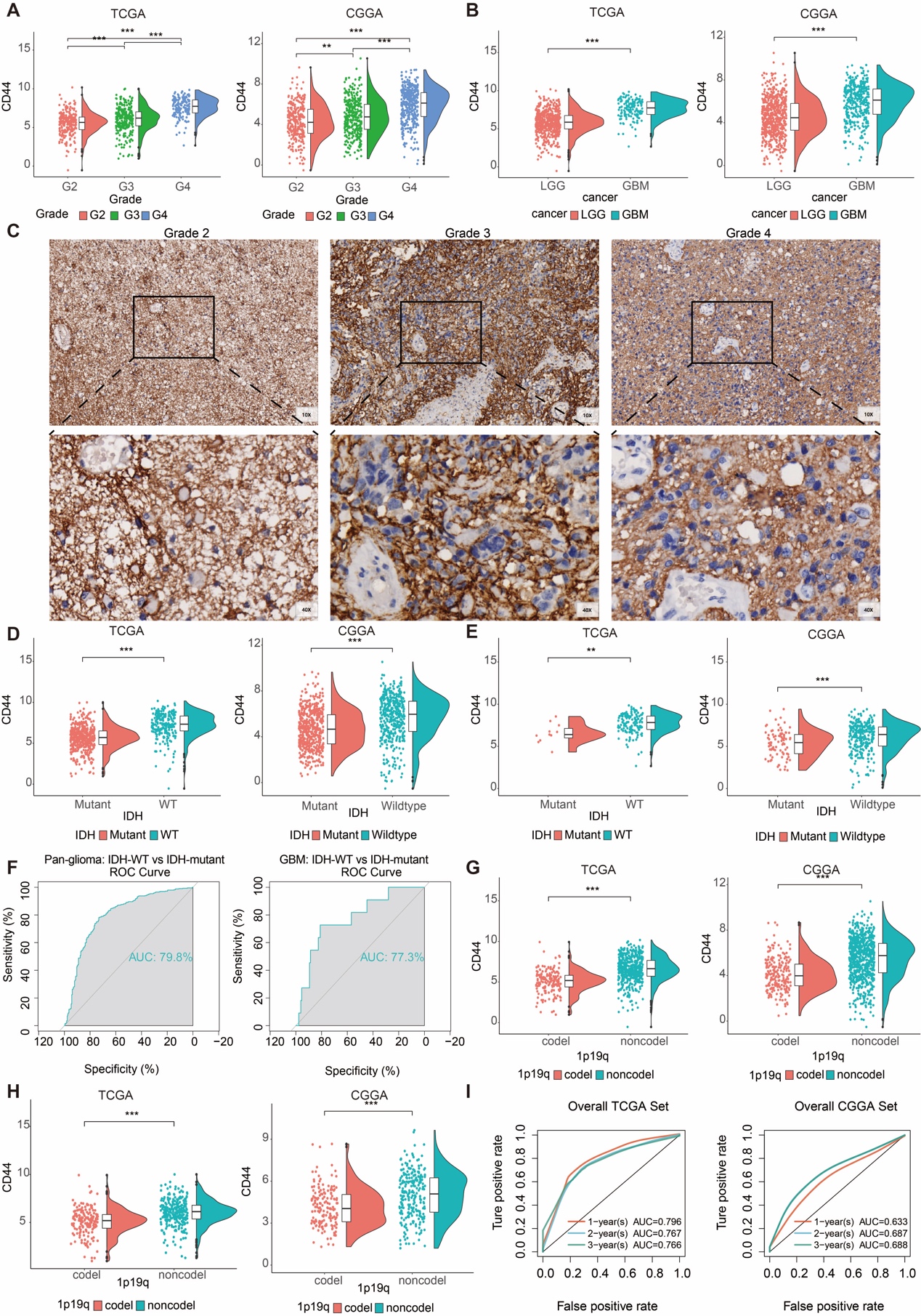


Fig.1. Up-regulated CD44 expression in aggressive gliomas. A. Analysis of CD44 mRNA levels (log2) in WHO grade II-IV gliomas from TCGA and CGGA. B. Analysis of CD44 mRNA levels (log2) in LGG and GBM from TCGA and CGGA. C. The representative images of CD44 in immunohistochemistry staining of different grades of glioma. D. CD44 expression was downregulated in the IDH mutant gliomas compared with the IDH wild-type gliomas in pan-glioma analysis from TCGA and CGGA. E. CD44 expression was downregulated in the IDH mutant gliomas compared with the IDH wild-type gliomas in GBM cases from TCGA and CGGA. F. Receiver operating characteristic (ROC) curve to assess sensitivity and specificity of CD44 expression as a predictive biomarker in IDH mutant gliomas in pan-glioma analysis and GBM cases from TCGA. G. CD44 expression was downregulated in the 1p/19q codeletion gliomas compared with the 1p/19q non-codeletion gliomas in pan-glioma analysis from TCGA and CGGA. H. CD44 expression was downregulated in the 1p/19q codeletion combined with IDH mutant gliomas compared with 1p/19q non-codeletion combined with IDH mutant gliomas in LGG cases from TCGA and CGGA. I. Receiver operating characteristic (ROC) curve to assess sensitivity and specificity of CD44 expression as a 3-years and 5-years prognosis biomarker in gliomas.


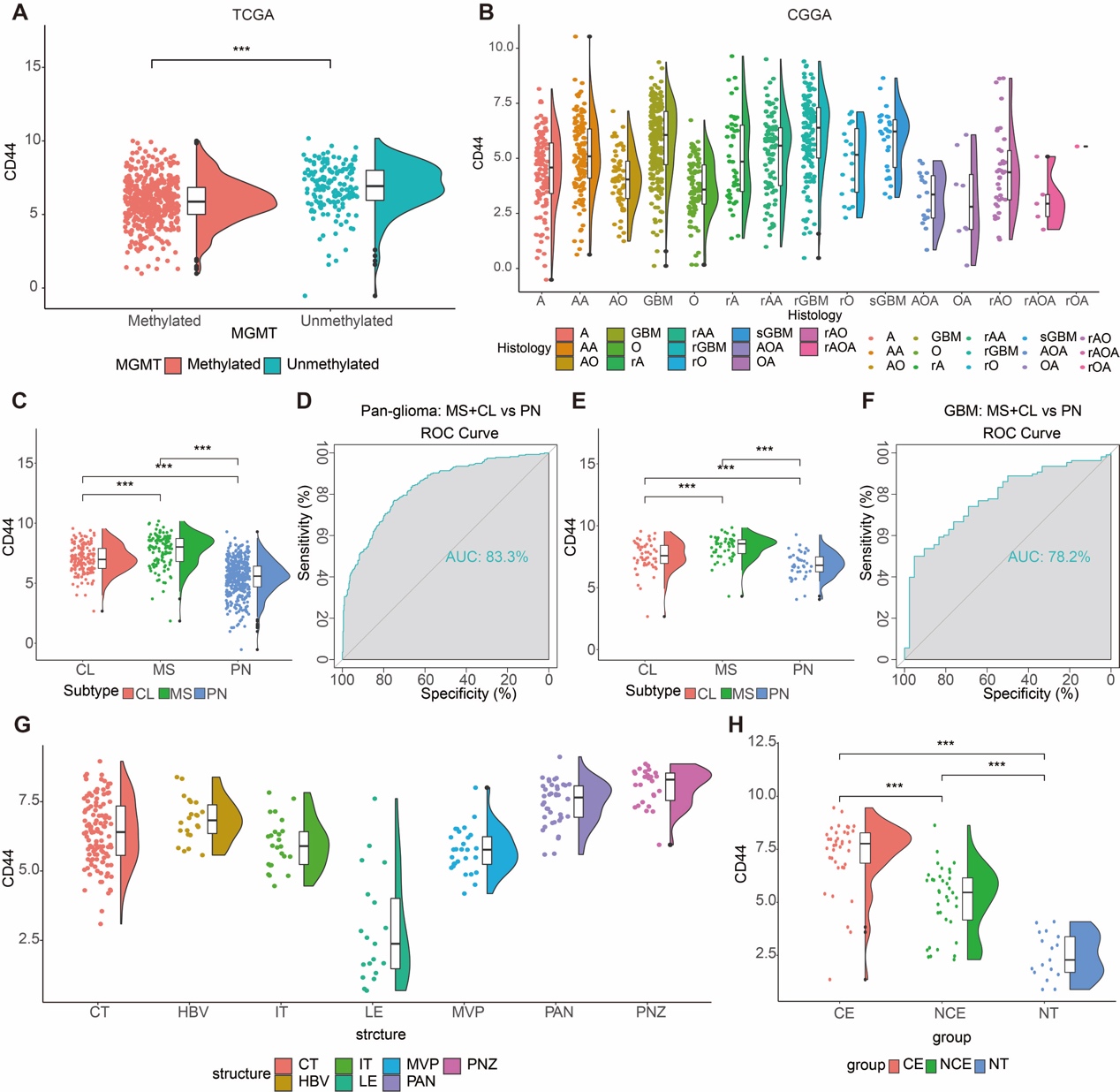


Fig.2. characteristics, expression pattern, and distribution of CD44 in gliomas. A. CD44 expression was downregulated in the methylated gliomas from TCGA. B. CD44 expression based on histopathologic classification. C. CD44 expression pattern in the TCGA molecular subtype in pan-glioma samples. D. ROC curves predicted CD44 as a biomarker of classical and mesenchymal subtype in pan-glioma samples. E. CD44 expression pattern in the TCGA molecular subtype in GBM samples. F. ROC curves predicted CD44 as a biomarker of classical and mesenchymal subtype in GBM samples. G. Analysis of CD44 expression using IVY GBM RNA-seq data based on anatomic structures: LE (Leading Edge), IT (Infiltrating Tumor), CT (Cellular Tumor), PAN (Pseudo palisading Cells Around Necrosis), PNZ (Perinecrotic Zone), MVP (Microvascular Proliferation), and HBV (Hyperplastic Blood Vessels). H. CD44 mRNA expression (log2) in the Gill dataset's disparate radio graphical regions of GBM and normal tissues. Radio graphical regions: CE (contrast-enhancing) regions, NCE (non-contrast-enhancing) margins, NT (normal tissues) areas.


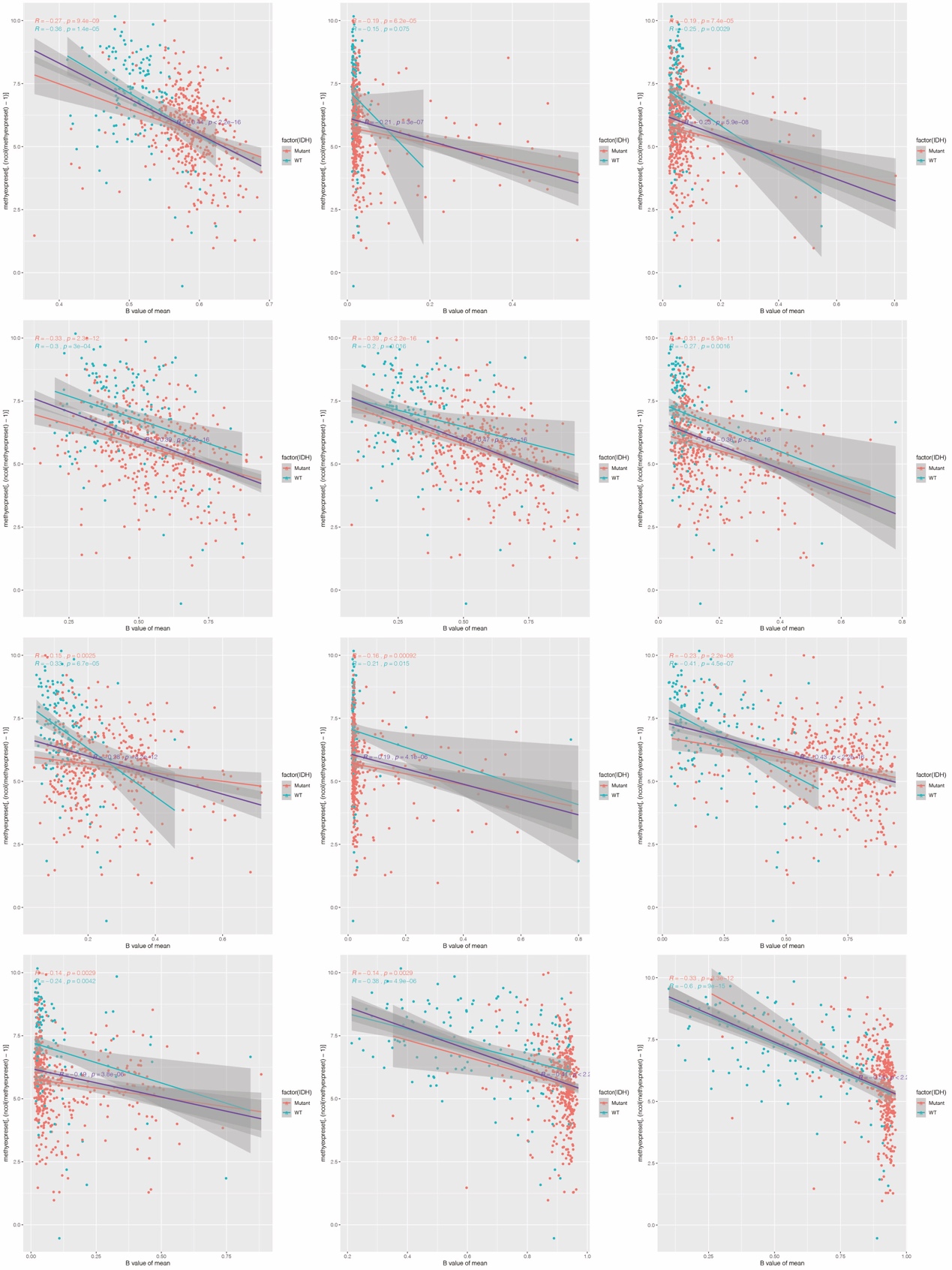


Fig.S3. Significant methylation probes associated with gliomas regarding CD44 expression.


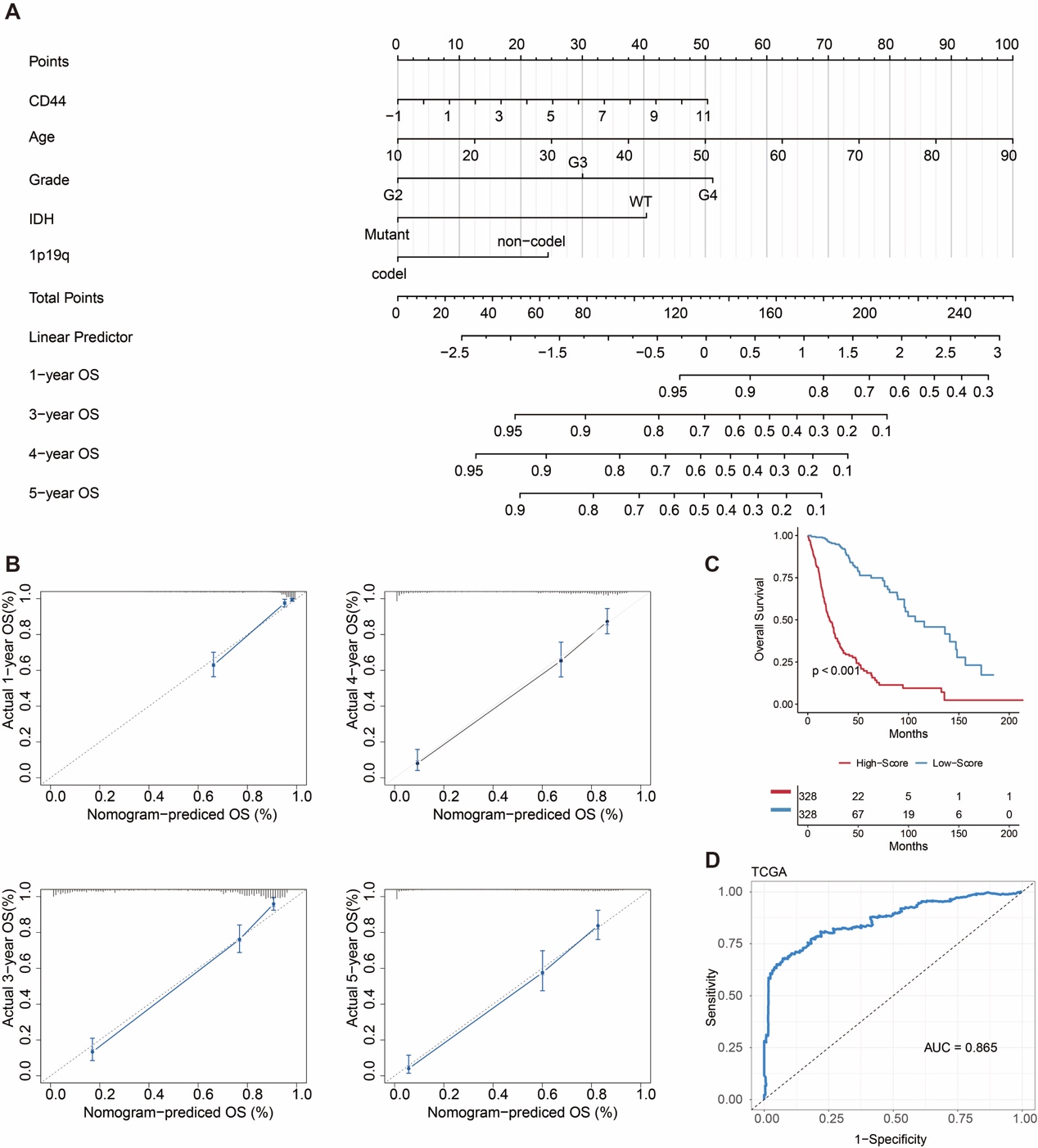


Fig.S4. Construction of the CD44-based nomogram. A. CD44 expression and clinicopathological risk factors were integrated to develop a prognostic nomogram model in the TCGA dataset. B. Calibration plots according to an ideal model. C. Kaplan-Meier survival curve between high and low-risk patients in the TCGA dataset. D. Receiver operating characteristic (ROC) curve to assess sensitivity and specificity of the prognostic nomogram model in the TCGA dataset.


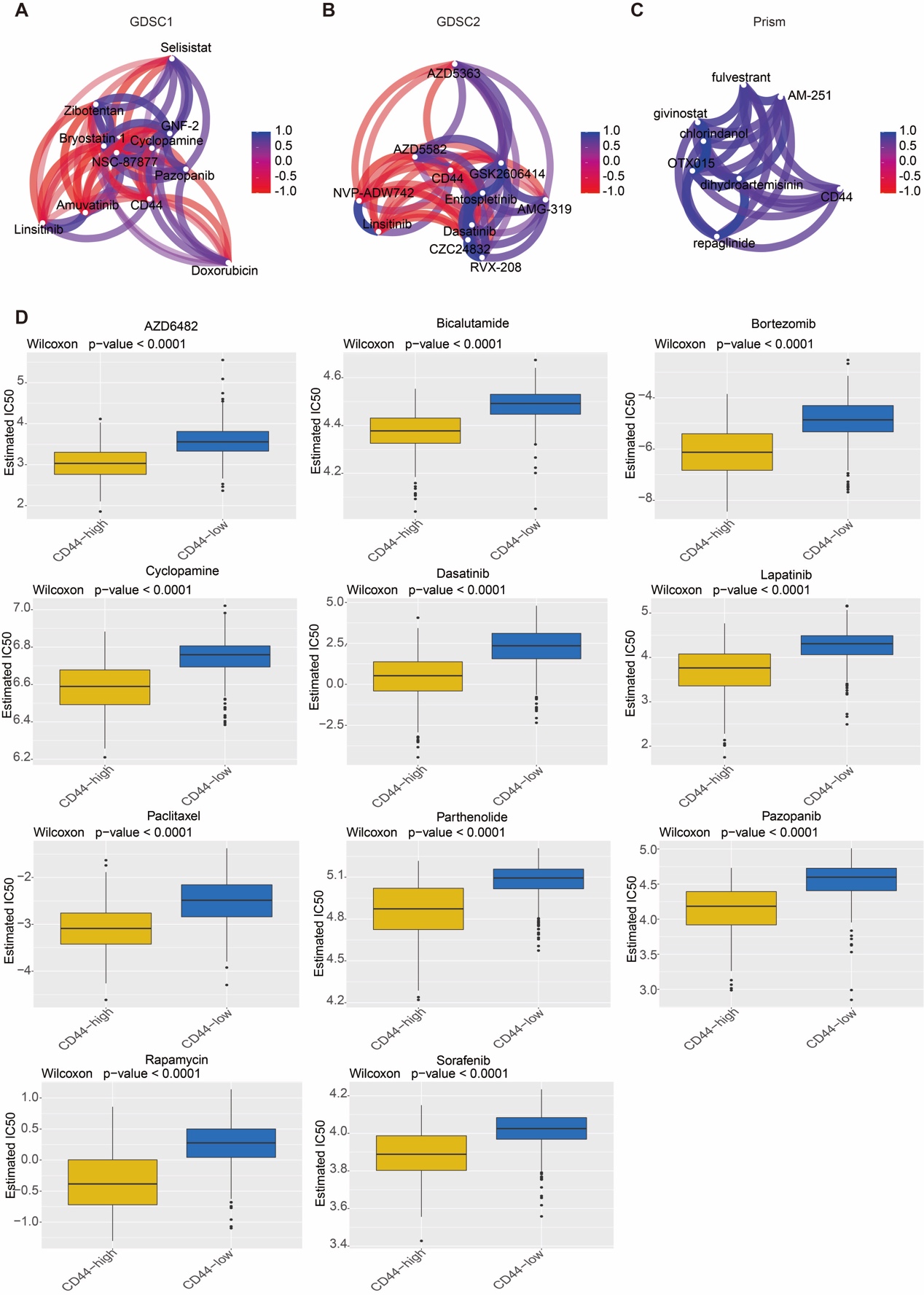


Fig.S5. Drug prediction based on PRISM and GDSC databases regarding CD44 expression.
